# Application of MIKE SHE to study the impact of coal mining on river runoff in Gujiao mining area, Shanxi, China

**DOI:** 10.1371/journal.pone.0188949

**Published:** 2017-12-21

**Authors:** Jianhua Ping, Shiyan Yan, Pan Gu, Zening Wu, Caihong Hu

**Affiliations:** School of Water Conservancy and Environment Engineering, Zhengzhou University, Zhengzhou, Henan Province, People’s Republic of China; University of A Coruña, SPAIN

## Abstract

Coal mining is one of the core industries that contribute to the economic development of a country but deteriorate the environment. Being the primary source of energy, coal has become essential to meet the energy demand of a country. It is excavated by both opencast and underground mining methods and affects the environment, especially hydrological cycle, by discharging huge amounts of mine water. Natural hydrological processes have been well known to be vulnerable to human activities, especially large scale mining activities, which inevitably generate surface cracks and subsidence. It is therefore valuable to assess the impact of mining on river runoff for the sustainable development of regional economy. In this paper, the impact of coal mining on river runoff is assessed in one of the national key coal mining sites, Gujiao mining area, Shanxi Province, China. The characteristics of water cycle are described, the similarities and differences of runoff formation are analyzed in both coal mining and pre-mining periods. The integrated distributed hydrological model named MIKE SHE is employed to simulate and evaluate the influence of coal mining on river runoff. The study shows that mining one ton of raw coal leads to the reduction of river runoff by 2.87 m^3^ between 1981 and 2008, of which the surface runoff decreases by 0.24 m^3^ and the baseflow by 2.63 m^3^. The reduction degree of river runoff for mining one ton of raw coal shows an increasing trend over years. The current study also reveals that large scale coal mining initiates the formation of surface cracks and subsidence, which intercepts overland flow and enhances precipitation infiltration. Together with mine drainage, the natural hydrological processes and the stream flows have been altered and the river run off has been greatly reduced.

## Introduction

Although coal mining industry has brought great economic benefits to human beings in the past centuries, more and more attention has been paid to the worsen water resources problems caused by the coal mining activities in recent years [1–6]. As an important water resource for production, living and ecology, river runoff in some areas has been rapidly attenuated or even discontinued due to the large-scale coal mining activity in recent years [7–11]. Therefore, to study the relationship between coal mining and river runoff has a significant value for both rational coal exploitation and sustainable utilization of regional water resources [12–18]. Zhang et al. [19] analyzed the annual runoff time series of Kuye River in Shenfu mining area by using a statistical method and discovered that the average annual runoff in the coal mining area was about a fifth of that prior to the coal mining activity. After further eliminating the influences of rainfall factors and conducting comparative analysis by using the multivariate regression analysis method, Zhang et al. [20] drew a conclusion that coal mining resulted in the decrease of average annual river runoff by 5.72 million m^3^, accounting for 32.2% of total runoff reduction. Wang [21] calculated the precipitation and flow data of Yangwu River over a 32 years’ period and concluded that the ratio coefficient of rainfall and base discharge decreased by 1/3 as a result of coal mining. Most recently, Zhou et al. [22] analyzed the correlation between runoff coefficient and coal mining activities of Kuye River and discovered that with the increase of coal mining, the runoff coefficient decreases. Although the above studies were able to provide a ballpark idea about the negative impact of coal mining toward river runoff, the statistical methods being used to establish multiple regression analysis are lacking of the consideration of a physical mechanism. Therefore, it is necessary to introduce a new approach to improve the precision of the quantitative analysis. In this paper, a comprehensive distributed hydrological modeling system MIKE SHE [23, 24] has been adopted to study the relationship between the coal mining and the river runoff in Gujiao area (Shanxi). The natural hydrological model of Gujiao in pre-coal mining period (1961–1980) is firstly constructed. Based on the ready-built model, the water cycle process without mining effect during the coal mining period (1981–2008) is simulated. By comparing the simulation results with the actual water process in the coal mining period (1981–2008), the influence of coal mining on river runoff of Gujiao is quantitatively calculated and analyzed based on the physical mechanism of water cycle and the formation path of runoff before and after coal mining activities take place.

## 1 Description of the study area

**Fig 1:**
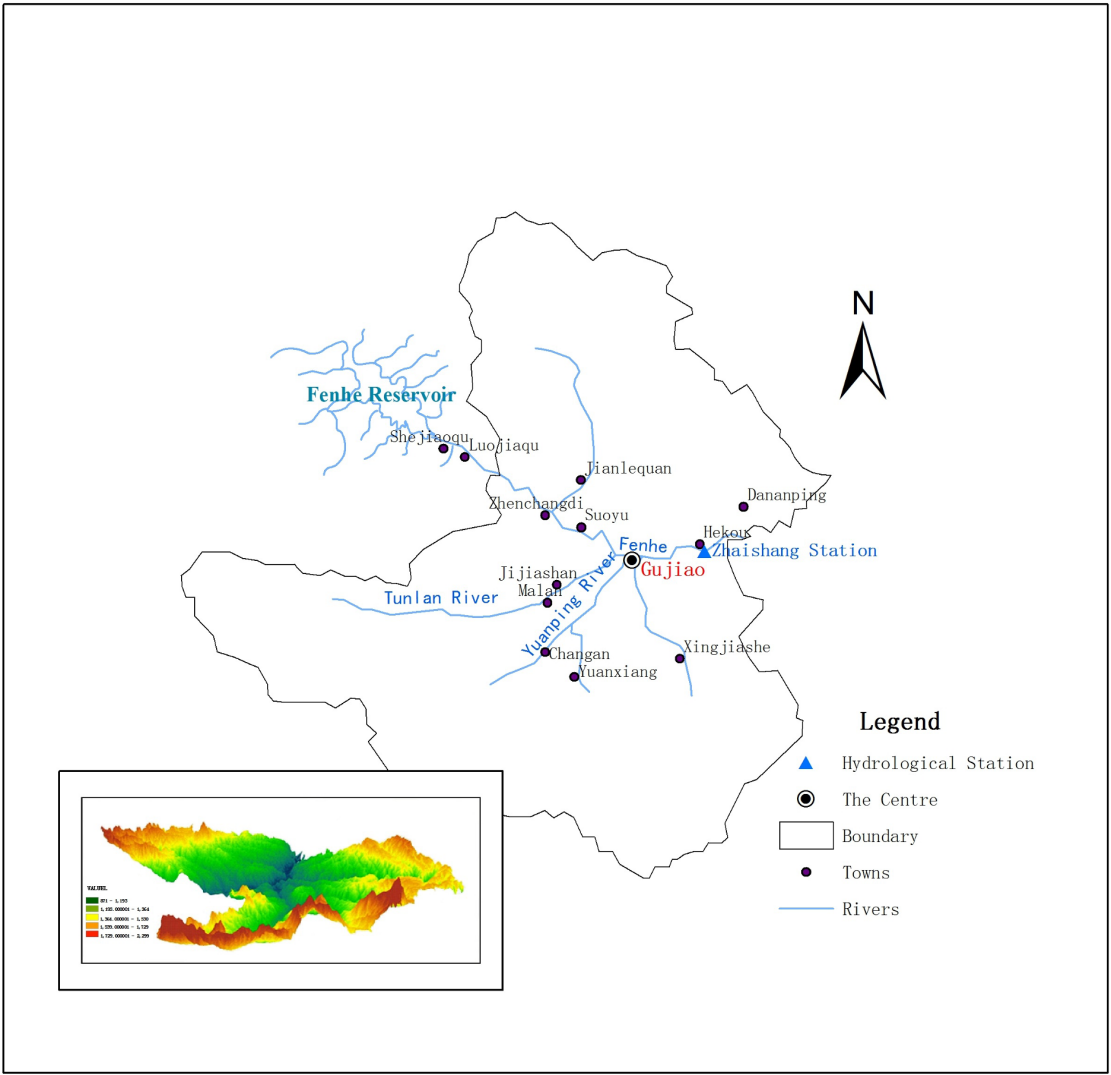
River systems and Topography of Gujiao.

**Fig 2:**
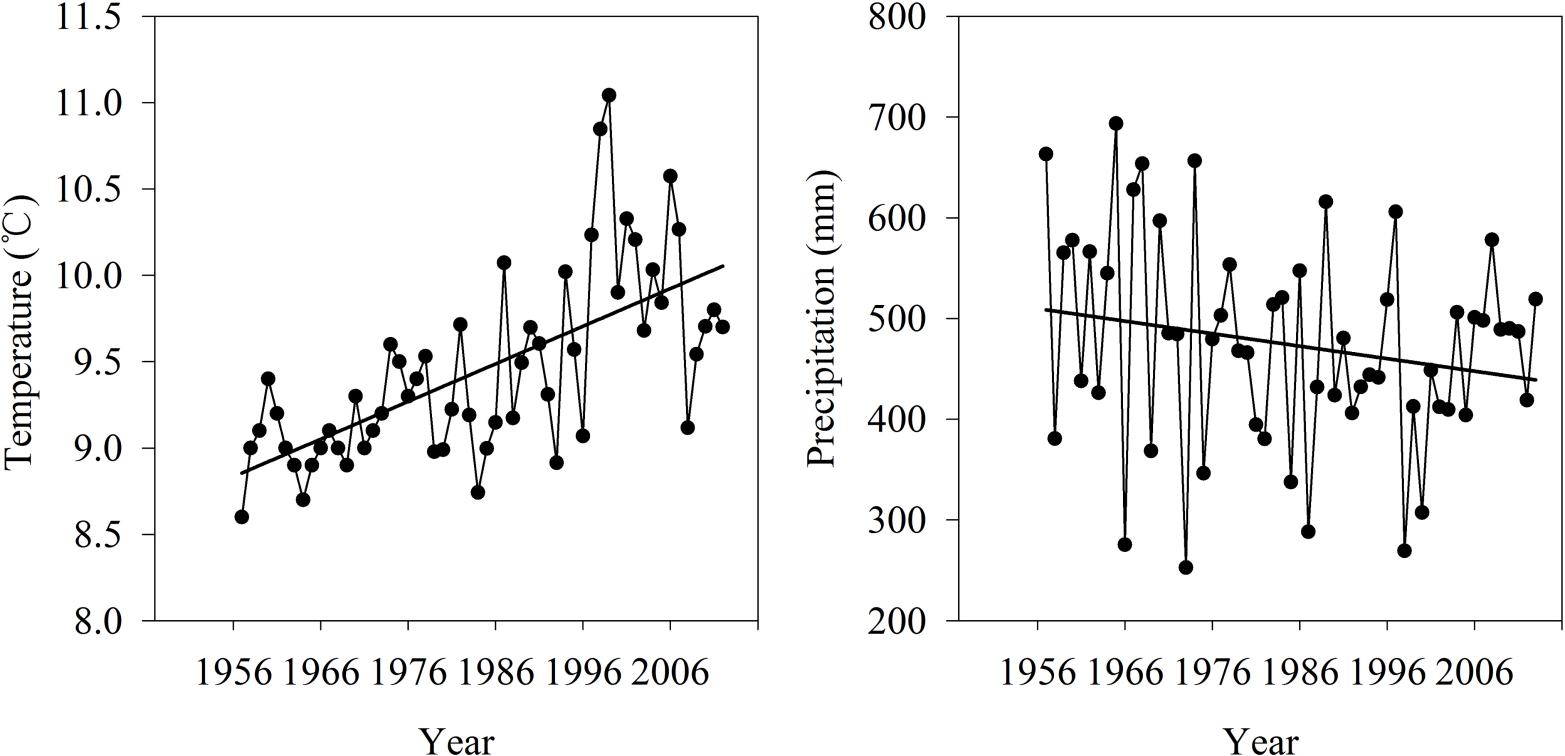
Temperature and precipitation in Gujiao from 1956 to 2012.

Gujiao, with a total area of 1584 km^2^, is located in the eastern foot of Liuliang Mountain’s middle section, to the west of Taiyuan city. Rivers in the study area form part of the Fenhe river system, at the Yellow River basin. It starts from Longweitou, runs eastward through the city, and drains out of the region near Saoshi, Hekou. There are four rivers with a catchment area of more than 100 km^2^: Tunlan River, Yuanping River, Dachuan River and Shizi River. Zhaishang station is the only hydrological station in Gujiao. The river map and the location of the hydrological station are listed in Fig 1. The average annual rainfall in Gujiao between 1961 and 1980 is 492.2 mm, and 415.1 mm after 1980, a reduction of 77.1 mm has taken place. The annual average temperature of the study area is 9.5°C. The variation trend of rainfall and temperature from 1955 to 2015 were presented in Fig 2. The terrain in the region is very complex, higher on all sides and lower in the center, with valleys extending in both vertical and horizontal directions. Mountains and hills account for 95.8% of the total area and river valleys account for 4.2%. The geology of Gujiao is also complex, including many faults, and the interaction between surface water and groundwater is intense.

There are abundant coal resources in the Gujiao coalfield, with proven reserves of 9.83 billion tons. The scale of coal deposit in Gujiao is also large, spreading a total area of 660 km^2^, which makes it one of the most important national coal chemical industry bases. Gujiao mining site is the only large scale mining site in the study area. The coal-bearing formations in the site are of Carboniferous Taiyuan formation and Permian Shanxi formation (2010). According to statistics data, there were limited, scattered and small scaled coals mining activities in the study area prior to1980, with an average annual raw coal production of 300 thousand tons. After 1980, large scale state-owned coal mines have been put into production and the annual raw coal yield grew rapidly since: the raw coal production in 1990 was 8.23 million tons, 9.565 million tons in 2000 and 20 million tons in 2008. In order to differentiate the significant change in the mining activities and better understand the impact of coal mining to the river runoffs, this study treats 1961–1980 as the natural, pre-coal mining period, and 1981–2008 as the coal mining period. Our work was approved by Bureau of Coal Geology of Shanxi Province, China and Shanxi Coal Geological Prospecting Institute of Hydrology, China. The study area is not privately owned or protected. The current research does not affect the protected species.

## 2 Materials and methods

Through the coupling of a one-dimensional surface water dynamics modeling system MIKE 11, MIKE SHE can be used to simulate various hydrological processes, the interaction among them, and establish a complete hydrological system [23, 25–28]. In order to construct the MIKE SHE model, the study area was divided into several regular grids, and individual grids are connected with associated physics equations [23, 29, 30]. This type of model has been proven to be able to resolve issues related to surface water and groundwater, through numerical simulation with finite difference methods [31–33].

### 2.1 Modeling framework

The module of the Gujiao model and corresponding calculation methods were summarized in Table 1. The parameters required for each module were specified in Table 2. Due to the limited available groundwater data, the linear reservoir method was adopted to construct the groundwater module in this paper.

**Table 1:** Hydrological process and corresponding methods of Gujiao MIKE SHE model.

Modules	Simulations	Theoretical methods
Overland Flow	Overland flow, Water depth, Sink Filling	Two dimensional Saint-Venant equations
Channel Flow (MIKE 11)	River runoff	One dimensional saint venant equations
Unsaturated Zone Flow And Evaporation	Unsaturated zone flow and water content, Evaporation, Infiltration and Groundwater recharge	2-layer UZ
Saturated Zone Flow	Ground water flow, Baseflow	Linear Reservoir

**Table 2:** The required information and parameters of Gujiao model.

Modules	The required information and parameters
Precipitation	Precipitation distribution
Overland Flow	Topographic map (DEM), Land use, Manning number, Detention storage distribution, Initial water depth
Channel Flow (MIKE 11)	River network distribution and characteristics, Geometric features of hydrologic monitoring cross section, Boundary conditions, Initial state
Unsaturated Zone Flow And Evaporation	Potential evaporation, Underground water level, Distribution and characteristics of soil, Saturated hydraulic conductivity of soil, Water content at saturation, Field capacity, Wilting point, Leaf evaporation index, Root depth
Saturated Zone Flow	Spatial distribution, The division of interflow reservoir and baseflow reservoir, Reservoir depth, Specific yield, Time constant

### 2.2 Data acquisition and processing

A complete MIKE SHE model requires properly formulated data and parameters [34, 35]. As a result, information such as meteorological data, terrain data and land use data need to be preprocessed. The parameters for river flow, overland flow and saturated flow module need to be determined by model calibration and verification (Table 2).

#### 2.2.1 Terrain

**Fig 3:**
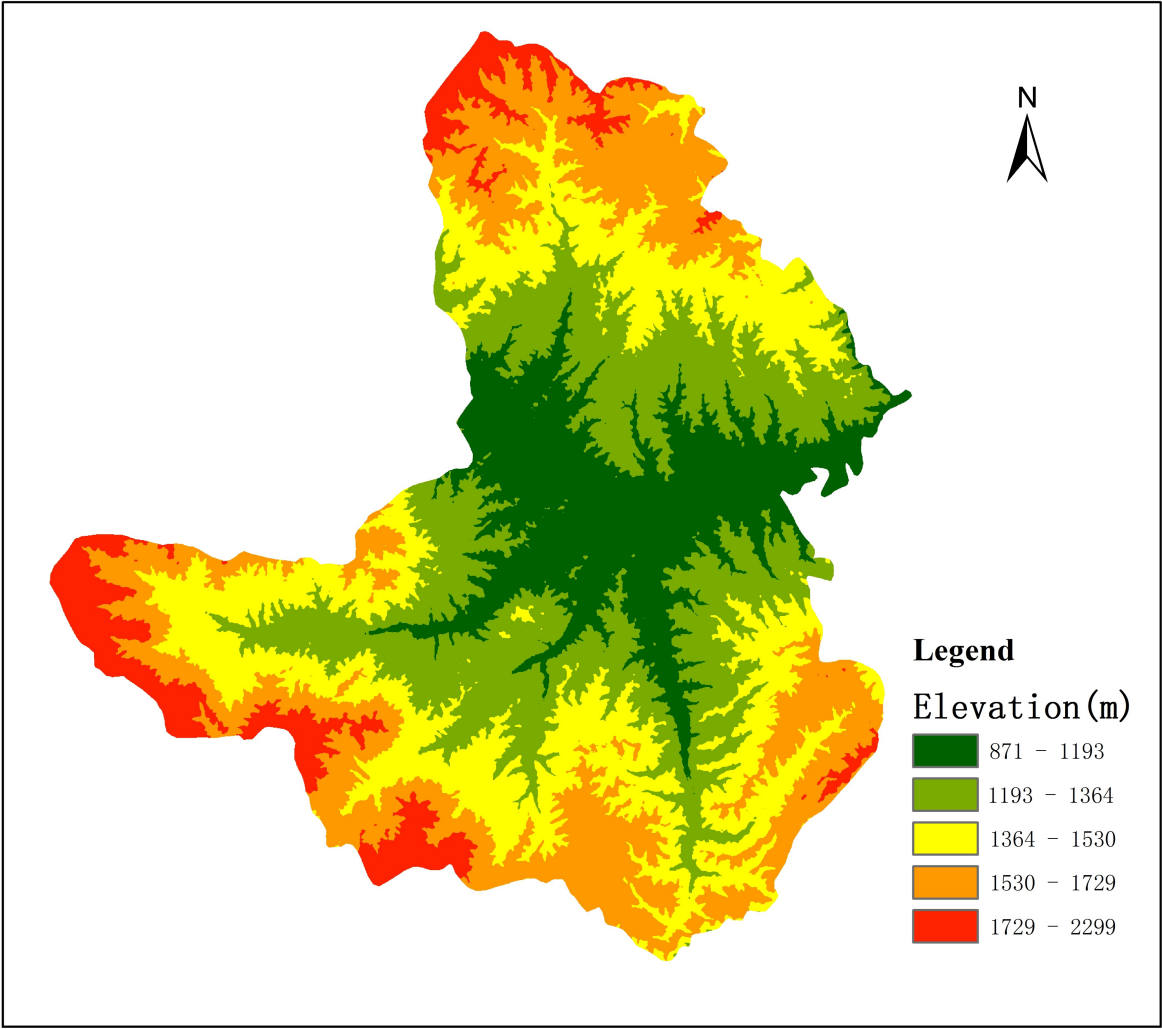
The DEM of Gujiao.

The boundary and scope were obtained by vectorizing the map (using ArcGIS 10.0), which was derived from Gujiao City’s groundwater resources evaluation reports. Next, topographic characteristics of the terrain were obtained by using digital elevation model (DEM), downloaded from the website of ASTER Global Digital Elevation Model (GDEM), with a resolution of about 30 m (Fig 3).

#### 2.2.2 Land use

**Fig 4:**
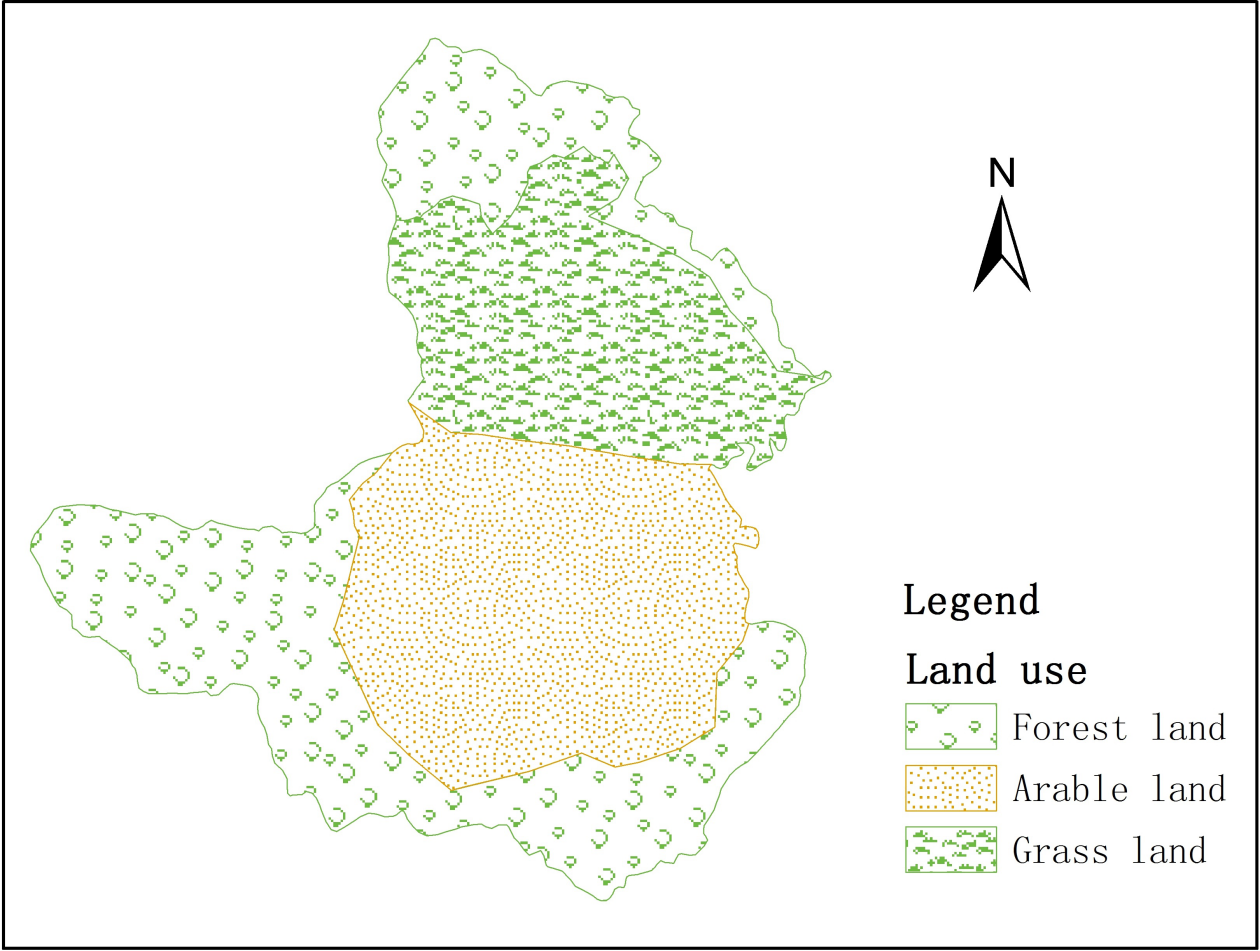
Land uses in Gujiao.

The land use data were obtained from land use vector graphics of Fenhe watershed, and converted into grid format (Fig 4). Land use module needed leaf area index (*LAI*), root depth (*RD*) and crop coefficient (*K*_*c*_) (Table 3). The data refer to Liu [36].

**Table 3:** The *LAI*, *RD* and *K*_*c*_ of the different land uses.

		Month											
		1	2	3	4	5	6	7	8	9	10	11	12
*LAI*	Arable land	0	0	0	0	0	0.1	0.1	0.2	0.4	0.3	0	0
	Forest land	0.1	0.1	0.1	0.2	0.5	1.5	1.6	1.6	1.5	1.5	0.5	0.1
	Grass land	0.1	0.1	0.1	0.2	1.2	1.5	1.6	1.7	2.5	2	0.5	0.1
*RD* (cm)	Arable land	15	15	15	15	15	15	15	15	15	15	15	15
	Forest land	1000	1000	1000	1000	100	100	100	1000	100	100	100	100
	Grass land	200	200	200	200	200	200	200	200	200	200	200	200
*K*_*c*_	Arable land	0	0	0	0	0	0.5	0.5	1	1.2	1	0	0
	Forest land	0.5	0.5	0.5	0.5	0.7	0.7	0.7	0.7	0.7	0.7	0.5	0.5
	Grass land	0.8	0.8	0.8	0.8	0.8	0.8	0.8	0.8	0.8	0.8	0.8	0.8

#### 2.2.3 Meteorology

Meteorological module needed precipitation and reference evapotranspiration. The specific process was as follow:

(1) Precipitation

Rainfall data obtained from 9 weather stations in Gujiao were used to calculate the regional rainfall by means of Thiessen polygon method (Table 4).

**Table 4:** Precipitation station and data sequence.

Number	Precipitation station	Data sequence before the coal mining	Data sequence after the coal mining
1	Changan	1971–1980	1981–2008
2	Meidonggou	1963–1980	1981–2008
3	Shuitou	1958–1980	1981–2008
4	Jialequan	1958–1980	1981–2008
5	Tuncun	1971–1980	1981–2008
6	Hekouzhen	1963–1980	1981–2008
7	Xingjiashe	1971–1980	1981–2008
8	Chakou	1958–1980	1981–2008
9	Geshang	1971–1980	1981–2008

(2) Reference evapotranspiration

Historical meteorological data including relative humidity (%), wind speed (m s^-1^), sunshine duration (h), maximum, minimum and average air temperatures (°C) were obtained from above nine weather stations to calculate the reference evapotranspiration with Penman-Monteith method.

#### 2.2.4 River

The river module needed river nets, cross section, boundary conditions and hydrodynamic parameters.

(1) River nets and cross section

**Fig 5:**
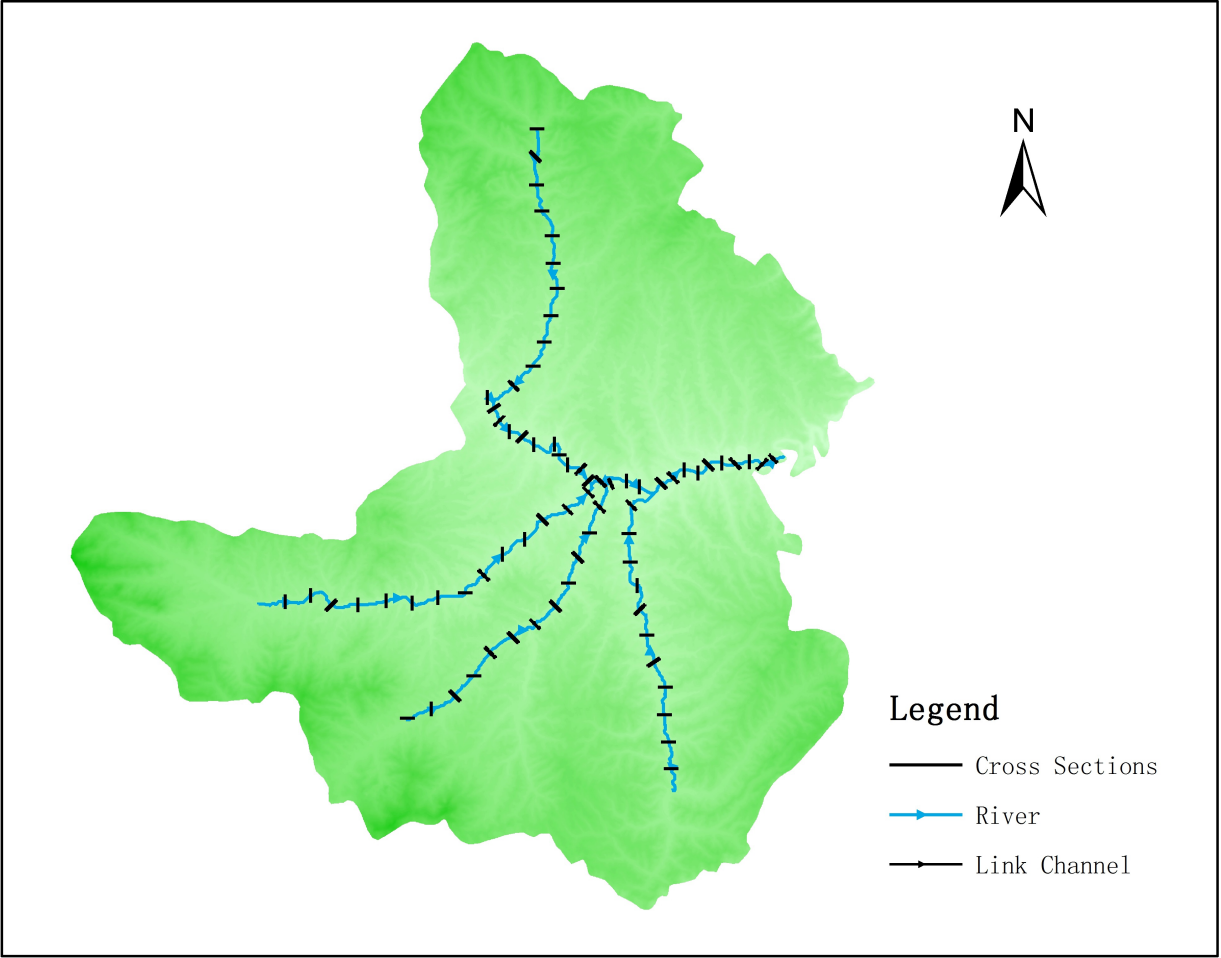
River nets and cross section.

The river nets were extracted by MIKE GIS. Based on the connection of main stream and tributary, we got the cross section (Fig 5).

(2) Boundary conditions and hydrodynamic parameters

In the model, the upstream boundary of Fenhe river was time sequence of flow. The upstream boundary of other tributaries were closed boundary, downstream boundary was the constant water level boundary. We determined the water level 2 m. The initial water depth of Fenhe river was 2 m. Each tributary was in dry condition, the initial water depth was 0 m, the manning coefficient of all rivers was 30.

#### 2.2.5 Overland flow

(1) Manning coefficient

Generally, the spatial distribution of manning coefficient depends on land uses, regional coefficient can be used without measurement. We treated the standard recommended value 8 as the initial value by the unified simple processing.

(2) Stagnant water deep

Stagnant water deep used the whole regional unified value, 10 mm.

#### 2.2.6 Unsaturated flow

**Fig 6:**
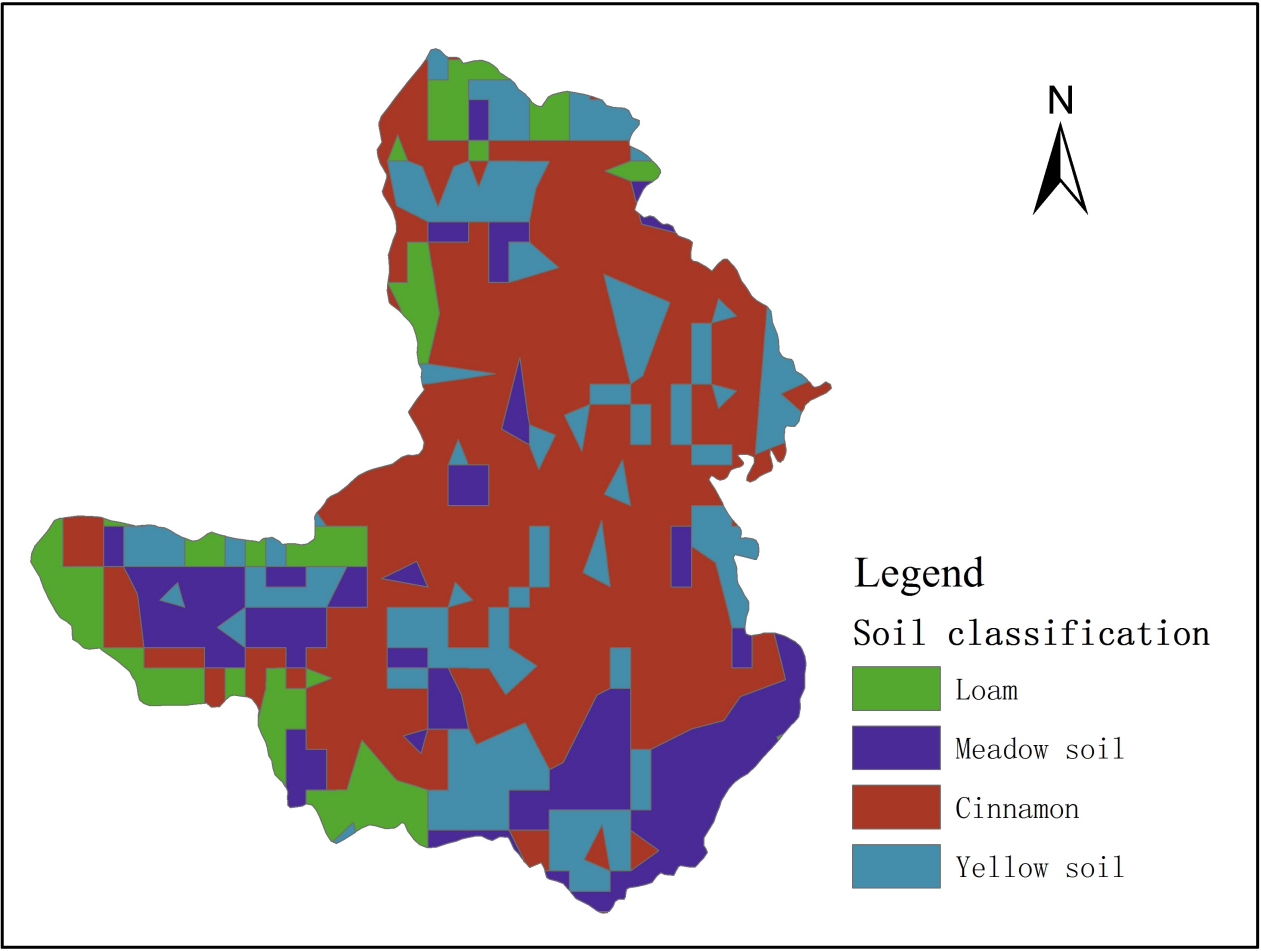
The distribution of soil types.

Unsaturated flow module needed soil parameters, such as, saturated soil water content, field capacity, wilting coefficient and saturated hydraulic conductivity coefficient (Table 5). Soil types determined the soil parameters, Gujiao had four kinds of soil types (Fig 6). The data were from local chronicles.

**Table 5:** Soil parameters for different soil types.

Soil types	Saturated soil water content (%)	Field capacity (%)	Wilting coefficient (%)	Saturated hydraulic conductivity (cm/s)
Brown earths	45	35	5	0.25
Cinnamon soils	45	35	5	0.11
Meadow soils	35	25	5	0.18
Cultivated loessial soils	35	2	5	0.21

#### 2.2.7 Saturated flow

(1) Interflow reservoir

**Fig 7:**
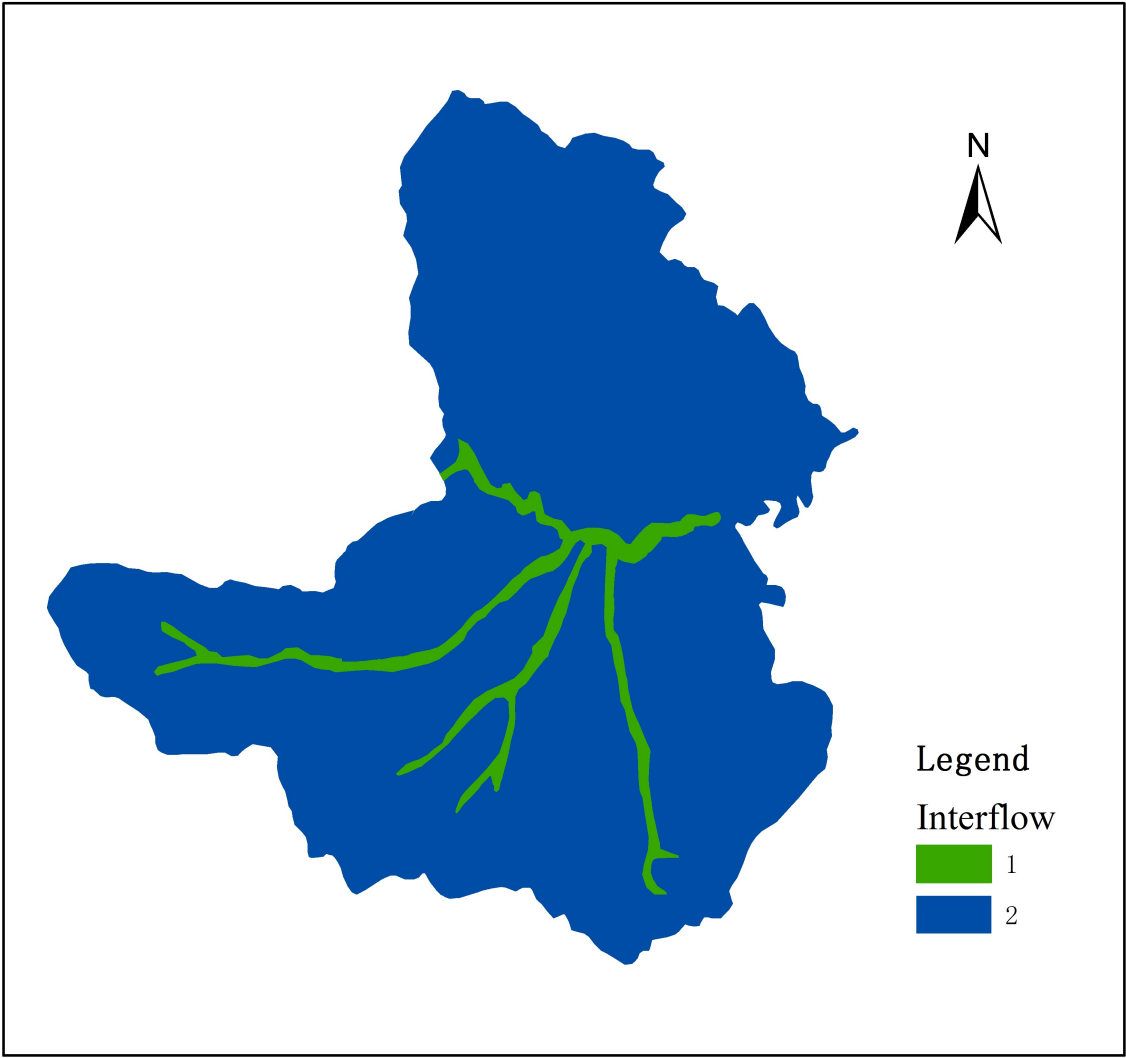
The distribution of interflow reservoirs.

In the model, the study area was divided into two interflow reservoirs, the upland interflow reservoir connected all the bedrock aquifer, the lower interflow reservoir connected all the alluvial aquifer (Fig 7). The input parameters were as follow: specific yield is 0.25; the initial depth is 4 m; time constant of interflow is 20 d; time constant of the seepage is 45 d.

(2) Baseflow reservoir

**Fig 8:**
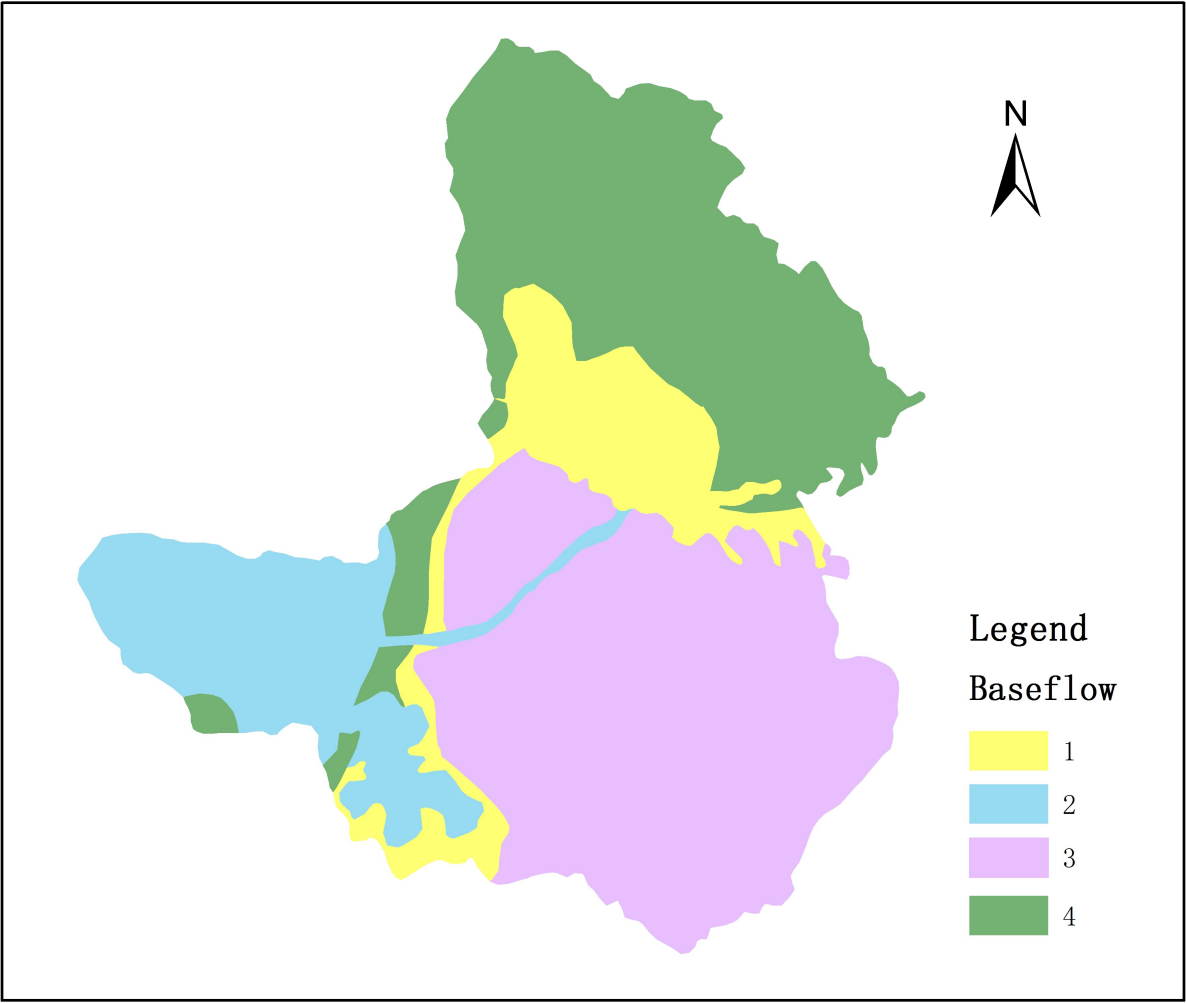
The distribution of baseflow reservoir.

The study area had five types of groundwater: the loose bed pore water carbonate rock, clastic rock fracture water, clastic rock fissure water and karst water of carbonate rock and metamorphic volcanic rock fissure water. The river around baseflow reservoir automatically connected with the reservoir. The study area is divided into four baseflow reservoir (Fig 8).

(3) Underground water level

The underground water level was not calculated, when underground water was simulated with the method of linear reservoir, which affected time and space distribution of groundwater recharge, but had no effect on the direction of subsurface flow. Therefore, we needed to define the underground water level clearly, which was boundary condition of the unsaturated zone.

The buried depth of Scuba diving in the pore water aquifer was shallow (3–5 m). Change of buried depth in the confined water level is bigger (>10 m). Therefore, the model parameter in the alluvium area was -5 m. Other areas were -10 m.

### 2.3 Calibration and validation

**Fig 9:**
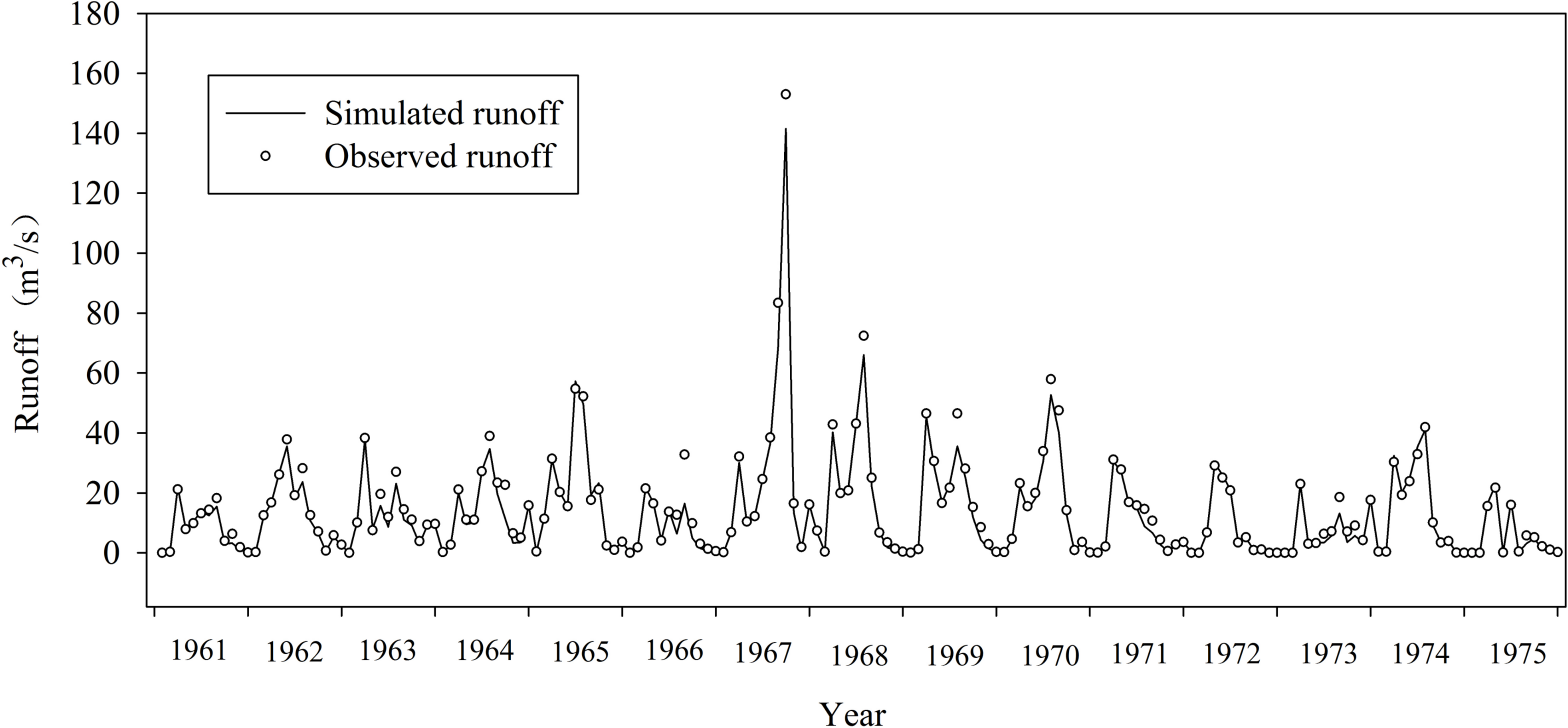
Monthly runoff simulation and scatter plot in calibration period (1961~1975).

**Fig 10:**
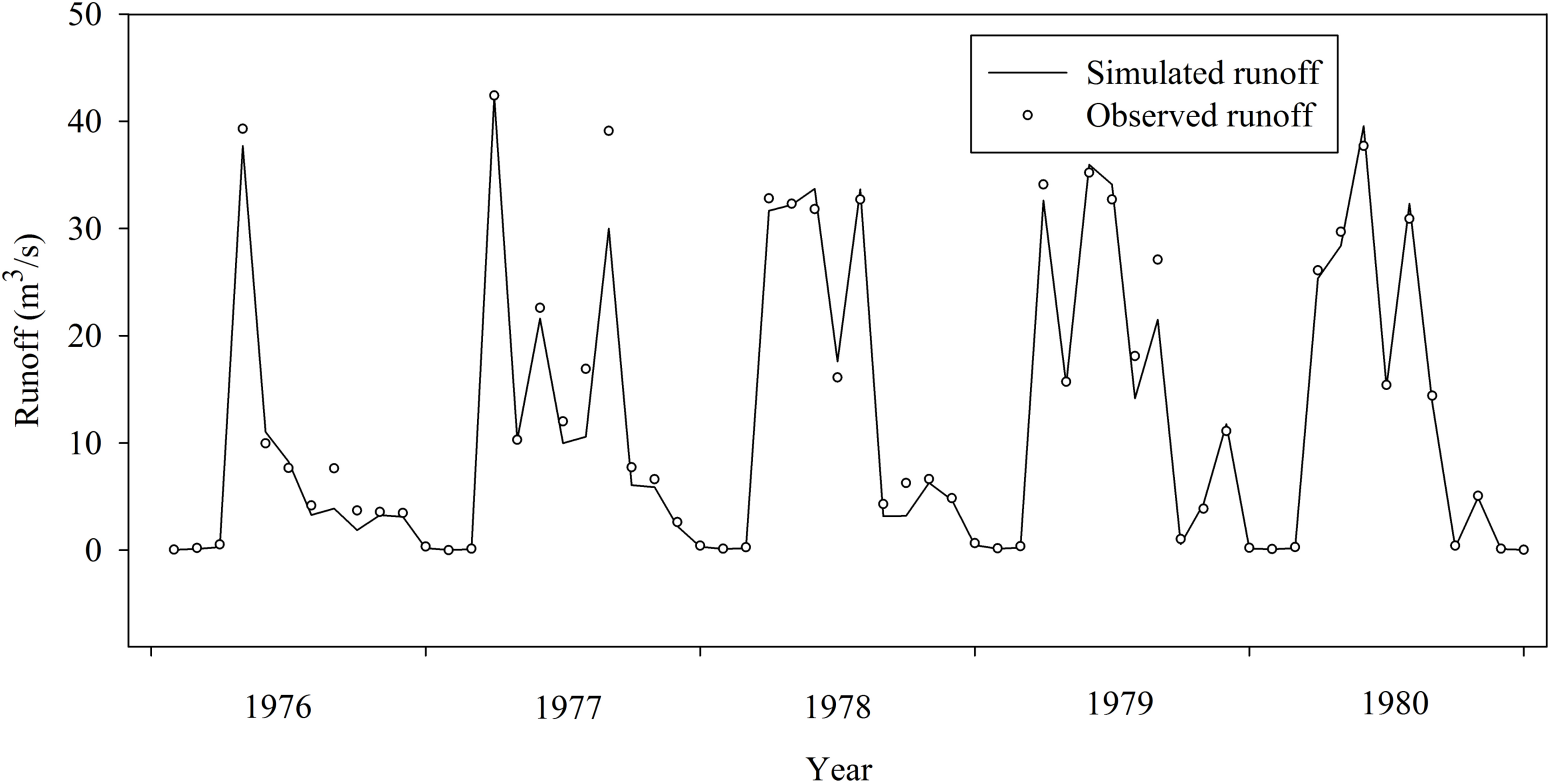
Monthly runoff simulation and scatter plot in validation period (1976~1980).

The monthly average runoff data of Zhaishang hydrological station for the period from 1961 to 1980 were used to validate the effectiveness of the simulation results. The period from 1961 to 1975 was set as the calibration period, and the validation period is from 1976 to 1980. Relative error index (RE), correlation coefficient index (R^2^) and Nash-Sutcliffe efficiency coefficient (ENS/NSE) were used to assess the simulation results. The evaluation results are listed in Table 6, and the simulation results are shown in Figs 9 and 10.

**Table 6:** Statistic indicators of model performance.

Index	Evaluation standard	Simulation period	
		Calibration period (1961~1975)	Validation period (1976~1980)
RE	<10%	-0.23	0.04
ENS	>0.6	0.87	0.94
R^2^	>0.5	0.93*	0.92*

* indicates that Kendall correlation coefficient pass the significance level 0.05.

Table 7 clearly indicates that both the calibration and evaluation periods satisfy all the compulsory criteria of the monthly model. Within the calibration period, the Nash-Sutcliffe efficiency coefficient is 0.87, with a relative error of -0.23 and the correlation coefficient 0.93. In comparison to the calibration period, data within the verification period have a better precision, with the Nash-Sutcliffe efficiency coefficient of 0.93, relative error 0.04 and correlation coefficient 0.92. Although certain data points are scattered off the calibration line (Fig 10), which are caused by the flood events, the overall trend has not significant differed from the observed monthly runoff. The shape of the runoff process line, the majority of flood peak values and peak times are also in good accord with the observed data. The result of the scatter plot also shows that various data points are uniformly distributed in a reasonable range with a high correlation. It is thus safe to say, the simulation results agree well with the observed data, and the established model with MIKE SHE could effectively simulate the natural water cycle process of Gujiao from 1961 to 1980.

**Table 7:** Comparison between simulated and actual annual average water process from 1981 to 2008.

Water Process (1981~2008)	Simulated results (mm)	Actual data (mm)	Variation (mm)	Study area (km^2^)	The variation in water quantity (million m^3^)
Precipitation	415		0	1584	0
Rainfall Infiltration	27.1	40	12.9		20.43
Leakage recharge	8.9	11.1	2.2		3.485
Surface runoff	12.3	10.9	1.4		2.218
Baseflow	28.7	13.4	15.3		24.23
River runoff	41	24.2	16.7		26.45

## 3 Results and discussion

**Fig 11:**
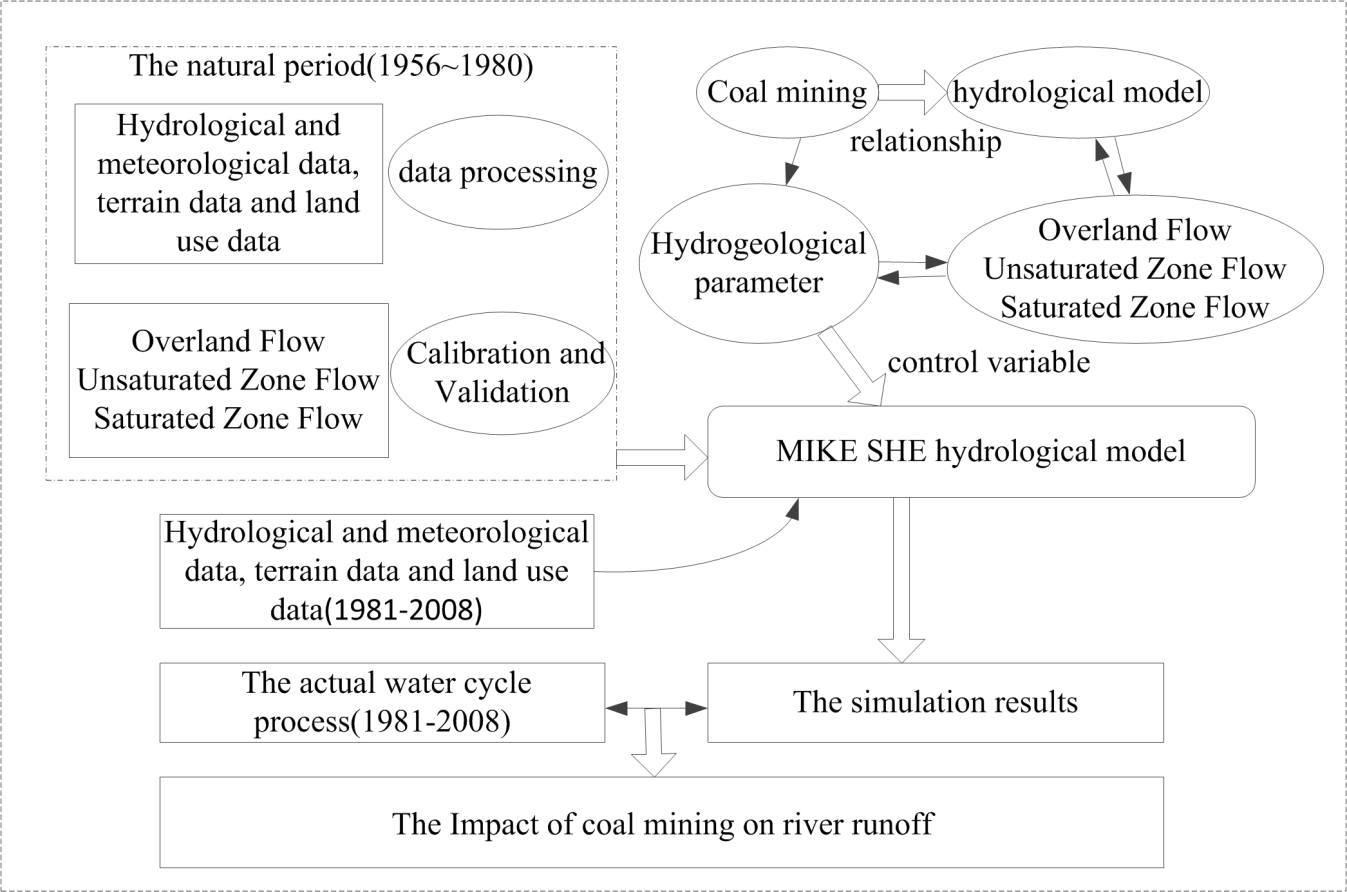
Technology roadmap.

As shown in Fig 11, the parameters identified by previously established model, including bed permeability coefficient, stagnant water deep and saturated hydraulic conductivity, could be used to reflect the natural structure of the underlying surface without mining effects. In order to simulate the water cycle processes with no effects from the mining activities for the mining period, both meteorological data and hydrological data from 1981 to 2008 were imported into the model, while keeping other parameters related to the overland flow, unsaturated flow and linear reservoir module of the natural hydrological model unchanged. Table 7 summaries and compares the simulation results to the actual water cycle process in coal mining period (1981~2008), which enables us to further analyze the influence coal mining on river runoff quantitatively by using the principle of water balance.

As shown in Table 7, there are great changes in rainfall infiltration, seepage, surface runoff and baseflow, etc. Combining the influence mechanism of coal mining on the water cycle and numerical simulation results obtained from MIKE SHE, the impact of coal mining on the surface runoff, baseflow, and river runoff were analyzed, respectively.

**Fig 12:**
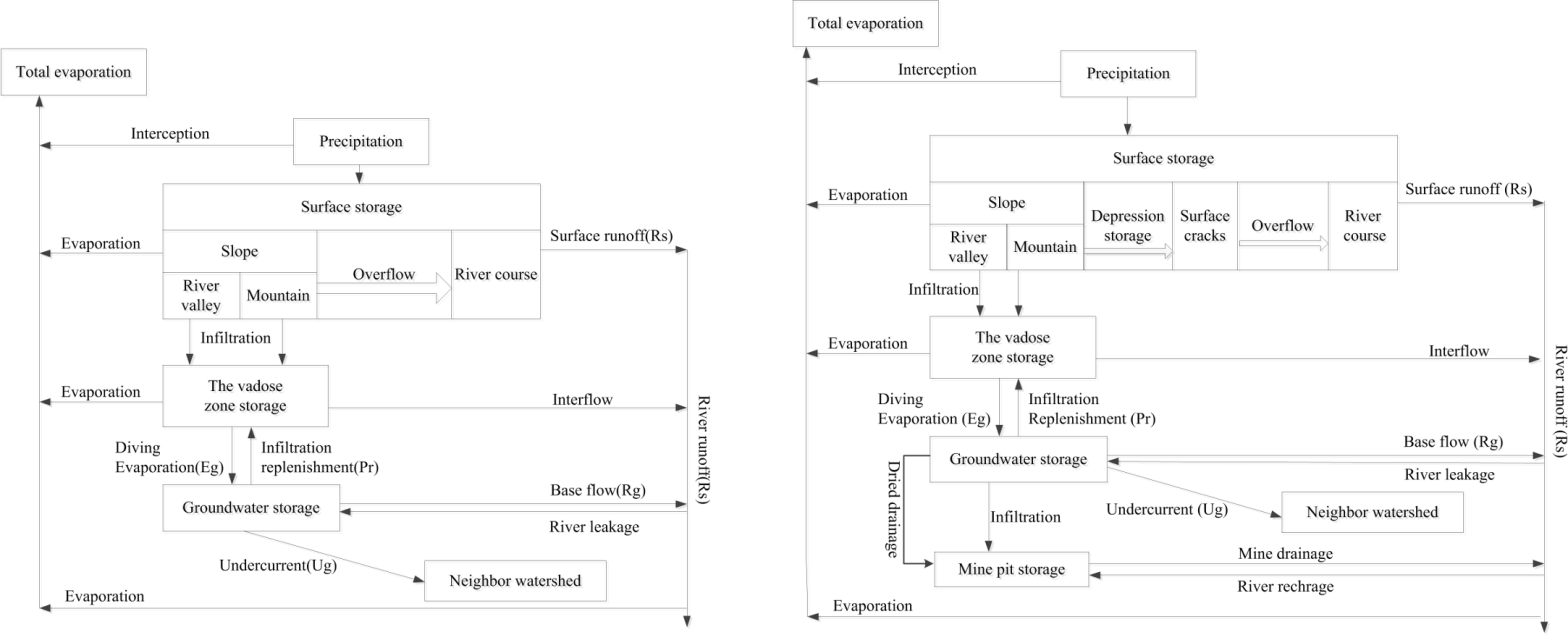
The hydrological cycle before and after large scale mining. (a) hydrological cycle before large scale coal mining. (b) hydrological cycle after large scale mining.

Previous researches have demonstrated that the activities of large-scale mining, blasting vibration and mine drainage in coal mining areas, with the impact of time and stress, would lead to geological structure reorganization, including the deformation of underground mined-out area, collapse and fracture in corresponding spatial [2, 15, 16]. On the other hand, the change of the geological environment would in return restructure the vadose zones and aquifers [37, 38]. Combining the effect of mine drainage, the water cycle process and quantity are also changed, including water recharge, runoff and discharge conditions of the surface water and groundwater [6, 18, 39], which further leads to the change of forming path and regularity of runoff in the mining period, as shown in Fig 12.

### 3.1 The influence of coal mining on surface runoff

Table 7 showed that the amount of simulated average annual rainfall infiltration in Gujiao from 1981 to 2008 is 27.1 mm, while the actual average annual rainfall infiltration is 40 mm, an increase of 12.9 mm, or about 20.43 million m^3^ in volume as a result of coal mining. At the same time, surface runoff is decreased by 1.4 mm. Similar observation has been reported by Gu [14]. The decrease of surface runoff can be due to the formation of collapse areas and cracks produced by mining [40, 41], which not only speeds up the infiltration of precipitated water, but also increases the volume of infiltration. In the process of runoff yield, part of the original overland flow is intercepted, which leads to the reduction of surface runoff that drains directly into the river [42].

### 3.2 The influence of coal mining on baseflow

Table 7 shows the influence of coal mining on the baseflow. It is clear that the simulated average annual baseflow within 1981–2007 was 28.7 mm, while the actual value is 13.4 mm. Therefore, coal mining activities in Gujiao in 1981–2008 result in the decrease of multi-year average river baseflow by 15.3 mm, or about 24.23 million m^3^ of water in volume. Table 7 also shows the simulated multi-year average leakage is 8.9 mm, or about 14.09 million m^3^ of water; while the actual multi-year average leakage of Fenhe River 11.1 mm in 1981–2008, about 17.66 million m^3^ of water. Therefore, coal mining activities cause the leakage of Fenhe River increased by 3.485 million m^3^ in Gujiao.

Precipitation infiltration is one of baseflow recharge sources [43]. The analysis above shows that the formation of coal mining subsidence cracks increases the precipitation infiltration, by means of the precipitation infiltration transformation from "piston" to "shortcut" [19]. This indicates that the presence of subsidence cracks increases the baseflow recharge sources. Baseflow is not only an important part of river runoff, but a double sense source of supplies, in which the river runoff by watercourse seepage recharges to groundwater [44, 45]. Due to the development of fractures under the action of coal mining, the hydraulic connections between different water sources are enhanced, causing the increase of Fenhe River’s seepage.

Under the natural state, baseflow excretes in form of evaporation and efflux [46, 47]. However, during the coal mining period, a large amount of groundwater would have been extracted. As a result, mine drainage becomes the main excretion way in the water cycle process, which drains away the river baseflow and destroys the existing motion path of the baseflow. Furthermore, it drops the underground water level and greatly reduces the surface runoff from groundwater recharge.

### 3.3 The influence of coal mining on river runoff

The effect of mining on the runoff flow rate in Gujiao was shown in Table 8. The impact of coal mining on river runoff from 1981 to 2008 is 16.7 mm in average, or about 26.45 million m^3^. Among them the decreased of surface runoff of Gujao was 1.4 mm, and the baseflow was 15.3 mm.

**Table 8:** The influence quantity on runoff caused by coal mine of Gujiao.

	Variation (mm)	Area (km^2^)	The influence quantity (million m^3^)	Raw coal production (million tons)	The influence quantity of each mining one ton of raw coal (m^3^/t)
Surface runoff	1.40	1584.00	2.22	9.23	0.24
Baseflow	15.30		24.23		2.63
River runoff	16.70		26.45		2.87

Statistic data shows the average annual production of raw coal in Gujiao is 9.228 million tons in the entire coal mining period (1981~2008). On the other hand, MIKE SHE model indicates that the annual reduction of runoff resulted from coal mining is 26.45 million m^3^. The influence of mining every ton of raw coal on river runoff of Gujiao can therefore be calculated, being 2.87 m^3^/t, of which 0.24 m^3^ was on surface runoff and the 2.63 m^3^ on baseflow. Coal mining has a greater impact on baseflow, which makes up 91.64% of the total reduction, while only 8.36% corresponds to a reduction of surface runoff. It is well known that surface runoff and baseflow are two main water supplies of river runoff. In the formation process of river runoff, the decrease of the both surface runoff and baseflow in Gujiao would inevitably lead to the decrease of river runoff in Gujiao, as a result of coal mining activities in the area.

### 3.4 The gradual impacts of coal mining on river runoff

The above sections have analyzed the relationships between coal mining and the runoffs in the mining period (1981~2008) as a whole. In fact, the influence of the coal mining to the underlying surfaces and water cycle process is rather slow and gradual. Therefore, it is necessary to divide the entire coal mining period into three sub-periods in order to better analyze the gradual effects of coal mining on river runoff. Table 6 lists both the statistical mining data in different sub-periods and the corresponding simulation results under the MIKE model.

**Fig 13:**
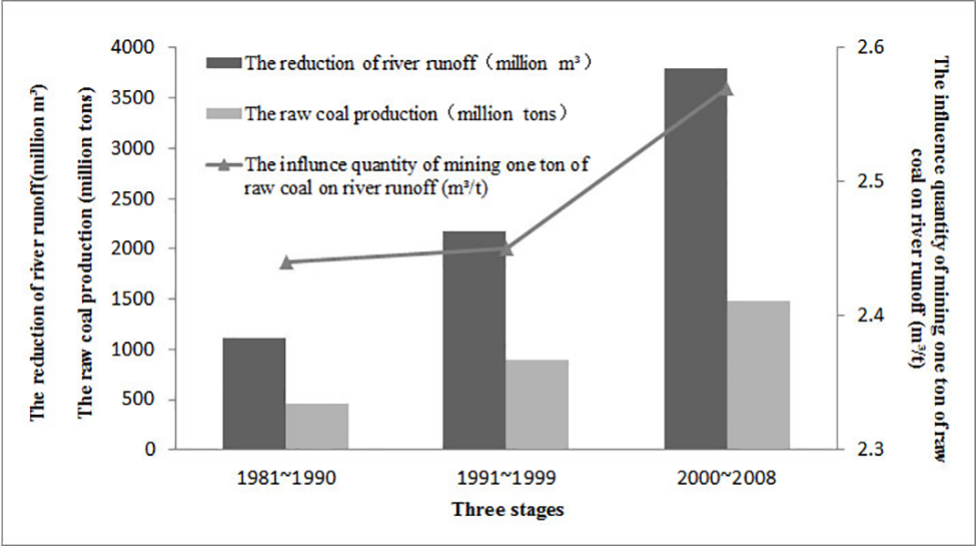
The influence of coal mining on river runoff in different stages.

The results indicate that, during 1981–1990, coal mining causes river runoff in Gujiao declined by an average of 11.13 million m^3^ per year, or 2.44 m^3^ for mining every ton of raw coal. During 1991–2000, coal mining causes river runoff in Gujiao declined by an average of 21.77 million m^3^ per year, or 2.45 m^3^ per ton of mined raw coal mined. For the period of 2001–2008, the annual river runoff deduction is 37.99 million m^3^, or 2.57 m^3^ per ton of mined raw coal mined. As shown in Table 9, the influence of mining every ton of raw coal to the river runoff is rather close for the first two sub-periods (2.44 m^3^ versus 2.45 m^3^), while there is a bigger jump for the period of 2001–2008. This could likely be the result of more coal mining and more serious damage being created by unjustified coal mining methods and technology used in this period, which leads to the destruction of the geological environment and water resources. The trend was shown in Fig 13.

**Table 9:** The influence of coal mining on river runoff in different stages.

Stages	Simulated runoff depth (mm)	Area (km^2^)	The simulated River runoff volume (million m^3^)	The actual River runoff volume (million m^3^)	The reduction (million m^3^)	Raw coal production (million tons)	The influence quantity of mining one ton of raw coal on river runoff (m^3^/t)
1981~1990	30.4	1584	48.15	37.02	11.13	4.56	2.44
1991~1999	49.7		78.67	56.90	21.77	8.90	2.45
2000~2008	37.6		59.49	21.50	37.99	14.78	2.57

## 4 Conclusions

Quantification analyses have revealed that mining one ton of raw coal leads to the decreasing of river runoff by 2.87 m^3^. The baseflow has been altered the most by the coal mining activities, which accounts for 91.64% of total river runoff reduction, while surface runoff made up only about 8.36%. The influence of coal mining on river runoff also presents an increasing trend with time, which is consistent with the intensifying mining activities. The current study further reveals that two new elements are joining the hydrological cycle process in the study area during coal mining period which are mine drainage and fissures. Coal mining changes the original water cycle path, which further changes the formation path of river runoff and leads to river runoff reduction. It is therefore clear to draw the conclusion that coal mining activities have played a significant role in the decrease of river runoff in Gujiao.

## Supporting information

S1 File Mini data set. (XLSX)
